# Iron-regulated metabolites produced by *Pseudomonas fluorescens* WCS374r are not required for eliciting induced systemic resistance against *Pseudomonas syringae* pv. *tomato* in Arabidopsis

**DOI:** 10.1002/mbo3.32

**Published:** 2012-08-24

**Authors:** Mohammad Djavaheri, Jesús Mercado-Blanco, C Versluis, J-M Meyer, L C Loon, Peter A H M Bakker

**Affiliations:** 1 Plant-Microbe Interactions, Department of Biology, Utrecht UniversityPadualaan 8, 3584 CH, Utrecht, The Netherlands; 2 Departamento de Protección de Cultivos, Instituto de Agricultura Sostenible (CSIC)Alameda del Obispo s/n, Apartado 4084, 14080, Córdoba, Spain; 3 Biomolecular Mass Spectrometry and Proteomics, Department of Chemistry, Utrecht UniversityPadualaan 8, 3584 CH, Utrecht, The Netherlands; 4 UMR7156 ‘Département Environnement, Génétique Moléculaire et Microbiologie’, CNRS, Université Louis Pasteur28 rue Goethe, F-67000, Strasbourg, France

**Keywords:** Induced systemic resistance, pseudobactin374, pseudomonine, salicylic acid

## Abstract

The plant growth-promoting rhizobacterium *Pseudomonas fluorescens* WCS374r produces several iron-regulated metabolites, including the fluorescent siderophore pseudobactin (Psb374), salicylic acid (SA), and pseudomonine (Psm), a siderophore that contains a SA moiety. After purification of Psb374 from culture supernatant of WCS374r, its structure was determined following isoelectrofocusing and tandem mass spectrometry, and found to be identical to the fluorescent siderophore produced by *P. fluorescens* ATCC 13525. To study the role of SA and Psm production in colonization of *Arabidopsis thaliana* roots and in induced systemic resistance (ISR) against *Pseudomonas syringae* pv. *tomato* (Pst) by strain WCS374r, mutants disrupted in the production of these metabolites were obtained by homologous recombination. These mutants were further subjected to transposon Tn*5* mutagenesis to generate mutants also deficient in Psb374 production. The mutants behaved similar to the wild type in both their Arabidopsis rhizosphere-colonizing capacity and their ability to elicit ISR against Pst. We conclude that Psb374, SA, and Psm production by *P. fluorescens* WCS374r are not required for eliciting ISR in Arabidopsis.

## Introduction

Iron is essential for the growth of almost all organisms but, in many environments, the amount of free iron is below 10^−7^ M, the concentration required by most bacteria for normal growth (Ratledge and Dover 2000). Bacteria have developed several strategies for the acquisition, solubilization, and transport of iron (Guérinot 1994). The most efficient mechanism of iron acquisition is the secretion of high-affinity iron-chelating compounds, so-called siderophores. The siderophores chelate iron in the extracellular environment, and the resulting ferric–siderophore complex is recognized by siderophore-specific membrane receptors, enabling uptake of Fe by the bacterial cells (Braun 2001).

Many bacteria produce more than one type of siderophore, as shown for *Enterobacter cloacae*, *Mycobacterium smegmatis,* and *Pseudomonas* spp. (Ankenbauer and Cox 1988; Meyer et al. 1992; Sokol et al. 1992; Loper et al. 1993; Buysens et al. 1996; Adilakshmi et al. 2000; Mercado-Blanco et al. 2001; Jones and Wildermuth 2011). Although considerable structural variation exists among the several hundreds siderophores chemically characterized at the present time, most can be classified as hydroxamates or catechols. Prototypical siderophores of these two classes are ferrichrome and enterobactin, respectively (Neilands 1981). The siderophores produced by fluorescent *Pseudomonas* spp. contain both hydroxamate and cathechol groups (Höfte 1993); these fluorescent siderophores have been named pyoverdines (Pvd). Moreover, some bacteria have the capacity to produce multiple siderophore-receptor proteins, enabling them to use not only their cognate siderophore complexes, but also those from other strains (Koster et al. 1993; Raaijmakers et al. 1994; Beare et al. 2003; Matthijs et al. 2009). Production of siderophores is one of the mechanisms by which microorganisms can antagonize each other (Bakker et al. 1988; Duijff et al. 1999; Loper and Henkels 1999). Such siderophore-mediated competition for iron between fluorescent *Pseudomonas* spp. and plant pathogens has been implicated in control of soilborne diseases (Bakker et al. 1986; Loper and Buyer 1991; Lemanceau et al. 1992; Höfte and Bakker 2007). Siderophores have also been implicated in induced systemic resistance (ISR) in plants (Maurhofer et al. 1994; Leeman et al. 1996; Meziane et al. 2005; Ran et al. 2005a; Bakker et al. 2007; Höfte and Bakker 2007; De Vleesschauwer et al. 2008). ISR is a state of enhanced defensive capacity developed by a plant when appropriately stimulated, and effective against a broad spectrum of both soilborne and foliar pathogens (Van Loon et al. 1998). Whereas siderophore-mediated suppression of plant diseases is restricted to the site where biocontrol bacteria and pathogens contact each other, ISR is effective throughout the plant and reduces disease incited by pathogens also at sites distant from the inducing bacteria.

Rhizobacteria-mediated induced resistance resembles pathogen-induced systemic acquired resistance (SAR), which is dependent on salicylic acid (SA) as a signaling compound (Ryals et al. 1996). Under iron-limited conditions, SA is synthesized by members of several bacterial genera, including *Azospirillum*, *Mycobacterium, Pseudomonas,* and *Yersinia*. In most cases, it has been suggested that SA acts as a siderophore in its own right (Ratledge et al. 1974; Meyer et al. 1992; Sokol et al. 1992; Visca et al. 1993; Adilakshmi et al. 2000). However, Chipperfield and Ratledge (2000) suggested that SA cannot bind ferric ions at pH values above 6, and according to these authors, SA itself would be unlikely to function as a siderophore.

Salicylic acid is a precursor or intermediate in the biosynthesis of certain types of siderophores, such as pyochelin (Cox et al. 1981), pseudomonine (Psm) (Mercado-Blanco et al. 2001; Sattely and Walsh 2008; Wuest et al. 2009), and yersiniabactin (Pelludat et al. 2003; Bultreys et al. 2006). SA synthesized by certain strains of *Pseudomonas aeruginosa* and *Pseudomonas fluorescens* has been suggested to be involved in the induction of systemic resistance against different plant pathogens (Leeman et al. 1996; De Meyer and Höfte 1997; Maurhofer et al. 1998; Audenaert et al. 2002). The ability of *P. aeruginosa* 7NSK2 to elicit ISR against *Botrytis cinerea* in bean was linked to SA production, as a mutant deficient in SA production of this strain was less effective; however, a role for pyochelin could not be excluded (De Meyer and Höfte 1997). For induced resistance elicited by *P. aeruginosa* 7NSK2, and *P. fluorescens* strains CHA0 and WCS374 against the bacterial pathogen *Pseudomonas syringae* pv. *tomato* (Pst) in Arabidopsis*,* Ran et al. (2005b) demonstrated that bacterially produced SA is not required.

Salicylic acid biosynthesis by bacteria occurs in a two-step reaction. In the first step, chorismate is converted to isochorismate by isochorismate synthase. This protein is encoded by the gene *pmsC* in *P. fluorescens* WCS374 (Mercado-Blanco et al. 2001) and *P. entomophila* L48 (Matthijs et al. 2009), which is homologous to the product of *pchA* in *P. aeruginosa* PA01 (Gaille et al. 2003). In the second step, isochorismate is converted to SA. This conversion is catalyzed by isochorismate-pyruvate lyase (Sattely and Walsh 2008; Wuest et al. 2009). The enzyme is encoded by gene *pmsB* in *P*. *fluorescens* WCS374 (Mercado-Blanco et al. 2001) and *P. entomophila* L48 (Matthijs et al. 2009), and by *pchB* in *P*. *aeruginosa* (Serino et al. 1995; Gaille et al. 2003). Künzler et al. (2005) have shown that PchB possesses both isochorismate-pyruvate lyase and weak chorismate mutase activities. In *Yersinia enterocolitica,* a single gene is involved, the encoded enzyme, Irp9, functions as a SA synthase by converting chorismate directly into SA (Pelludat et al. 2003). Gene *Irp9* does not contain sequences similar to *pchB* or *pmsB*.

Mercado-Blanco et al. (2001) found that genes *pmsB* and *pmsC* of WCS374 are part of an operon, *pmsCEAB*, that is involved in the biosynthesis of SA and Psm under iron-limiting conditions. Heterologous expression of *pmsB* and *pmsC* was achieved in *Pseudomonas putida* and *Escherichia coli* cells. In *E. coli*, deletions affecting *pmsC* diminished SA production, whereas deletion of *pmsB* abolished it completely. The *pmsB* gene enabled *E*. *coli* to produce low levels of SA when expressed under control of the *lacZ* promoter. Mass spectrometric and NMR analyses indicated that Psm is composed of SA, cyclothreonine, and histamine. The translational product of *pmsA* showed high homology with pyridoxal phosphate-dependent histidine decarboxylases, indicating that this gene must be involved in the synthesis of the histamine moiety. PmsE showed similarities to proteins involved in the biosynthesis of siderophores such as enterobactin (EntE), pyochelin (PchD), vibriobactin (VibE), and yersiniabactin (YbtE), and is a nonribosomal peptide synthetase (NRPS) (Sattely and Walsh 2008; Wuest et al. 2009). In *P. fluorescens* WCS374 and *P. entomophila* L48, 15 ORFs that encode pseudomonine biosynthesis and putative transport genes with identical organization have been described (Matthijs et al. 2009).

Whereas *P. fluorescens* WCS374 induces systemic resistance in radish (Leeman et al. 1995), it normally does not do so in Arabidopsis (Van Wees et al. 1997; Ran et al. 2005b). Exogenously applied SA induces resistance in both plant species. Compared to other SA-producing fluorescent *Pseudomonas* spp. strains, WCS374 produces relatively high amounts of SA in vitro (Leeman et al. 1996). However, it is not clear whether this strain produces SA in the rhizosphere, and the apparent inability of WCS374 to induce resistance in Arabidopsis actually suggests that SA is not released. It might be that SA is produced in the rhizosphere, but all of it is channeled into Psm, comparable to the suggestion of Audenaert et al. (2002) that in *P*. *aeruginosa* 7NSK2, SA is channeled into pyochelin.

Effective root colonization by *Pseudomonas* spp. is a prerequisite for successful suppression of soilborne plant diseases (Lugtenberg et al. 2001; Mercado-Blanco and Bakker 2007). A minimum bacterial cell density is required for both microbial antagonism and induction of systemic resistance (Raaijmakers et al. 1995). Efficient and specific iron-supplying systems appear to be important for fluorescent pseudomonad fitness and competitiveness (Bakker et al. 1988; Loper and Henkels 1999). In order to unravel the role of SA and Psm biosynthesis in the ecology of *P. fluorescens* WCS374 in the rhizosphere, mutational analysis of the genes *pmsA* and *pmsB* was performed. Mutations in the biosynthesis of Pseudobactin374 (Psb374), the pyoverdine produced by WCS374r, in the background of the wild-type strain and the *pmsA* and *pmsB* mutants enabled us to study the significance of these three iron-regulated metabolites in the fitness of WCS374 in the rhizosphere of Arabidopsis. In contrast to experiments in which ISR is studied (Pieterse et al. 1996; Van Wees et al. 1997; Ran et al. 2005b), very low population densities of the bacteria were applied in root colonization studies to allow active growth of the introduced bacteria, thereby possibly enhancing differences between the wild-type strain and the mutants. When introduced at such low densities, WCS374 did effectively elicit ISR against Pst, allowing us to study the involvement of the iron-regulated metabolites of this *P. fluorescens* strain in ISR.

## Materials and Methods

### Bacterial strains and growth conditions

*Pseudomonas fluorescens* WCS374 was originally isolated from the rhizosphere of potato (Geels and Schippers 1983), and WCS374r, a spontaneous rifampicin-resistant mutant, was used in further investigations. Psb374-negative mutants have been characterized previously (Weisbeek et al. 1986; Leeman et al. 1996). All strains and plasmids used are listed in Table 1. *Pseudomonas fluorescens* and *Pseudomonas putida* strains were grown at 28°C in King's medium B (KB) (King et al. 1954), and *E. coli* and *P. aeruginosa* strains were grown at 37°C in Luria–Bertani (Miller 1972) and KB medium, respectively. All plasmids were propagated in *E*. *coli* DH5α at 37°C. Antibiotics were added at the following concentrations (μg/mL) when needed: ampicillin (Amp), 100; kanamycin (Km), 25; nalidixic acid (Nal), 25; rifampicin (Rif), 200; and tetracycline (Tc), 20 (to stop growth of *E. coli*) or 40 (for *Pseudomonas*).

**Table 1:** Bacterial strains and plasmids used in this study and their relevant characteristics

Strains/plasmids	Characteristics	Source
***Escherichia coli***		
DH5α	*recA1 endA1 f80d lacZ dam-15*	Clontech
HB101	*pro leu thi lacY endA recA hsdR hsdM* Sm^r^	Boyer and Roulland-Dussoix 1969
S17-1	*thi pro recA hsdR hsdM* RP4-2-Tc,Mu-Km, Tp^r^ Sm^r^	Simon et al. 1983
***Pseudomonas***		
*P. fluorescens* WCS374	Wild type; Psb^+^ Psm^+^ SA^+^, Nal^r^	Geels and Schippers 1983
WCS374r	Spontaneous rifampicin-resistant mutant of WCS374; Psb^+^ Psm^+^ SA^+^, Rif^r^	Geels and Schippers 1983
374-02	WCS374 Tn*5* mutant lacking pseudobactin; Psb^−^ Psm^+^ SA^+^, Km^r^	Weisbeek et al. 1986
374-08	WCS374 Tn*5* mutant lacking pseudobactin; Psb^−^ Psm^+^ SA^+^, Km^r^	Mercado-Blanco et al. 2001
4A-1	Exchange mutant of *pmsA* homolog of WCS374r; Psb^+^ Psm^−^SA^+^, Km^r^ Rif^r^	This study
AT-12	Tn*5* mutant of 4A-1; Psb^−^ Psm^−^ SA^+^, Km^r^ Rif^r^ Tc^r^	This study
4B-1	Exchange mutant of *pmsB* homolog of WCS374r; Psb^+^ Psm^−^ SA^−^, Km^r^ Rif^r^	This study
BT-1	Tn*5* mutant of 4B-1; Psb^−^ Psm^−^ SA^−^, Km^r^ Rif^r^ Tc^r^	This study
**Plasmids**		
pE1	12260-bp *Eco*RI fragment from pMB374-07 in both orientations in pGEM-3Z	This study
pE4	3268-bp *Eco*RI fragment from pMB374-07 in both orientations in pGEM-3Z	This study
pGEM-3Z	Cloning and transcription vector; Amp^r^	Promega
pJMSal-10	pGEM-3Z with 0.7-kb *Eco*RI – *Pst*I fragment containing *pmsB* gene	Mercado-Blanco et al. 2001
pJMSal-20	pGEM-3Z with 1.7-kb *Eco*RI – *Hin*dIII fragment containing *pmsAB* genes	Mercado-Blanco et al. 2001
pJMsal-10::Km	pJMSal-10 containing a Km cassette in *Mlu*I site	This study
pJMsal-20::Km	pJMSal-20 containing a Km cassette in *Sal*I site	This study
pJMpmsA::Km	*pmsA*::Km fragment subcloned from pJMsal-20::Km in pSUP202	This study
pJMpmsB::Km	*pmsB*::Km fragment subcloned from pJMsal-10::Km in pSUP202	This study
pJQ18	pSUP5011 derivative; carries Tn*5*-Mob-Tc^r^	Hynes et al. 1989
pMB374-07	28 kb from WCS374 in pLAFR1; carries SA and psm biosynthesis genes	Mercado-Blanco et al. 2001
pRK2013	ColE1 replicon, helper plasmid; Km^r^ tra	Figurski and Helinski 1979
pSUP202	Amp^r^ Chl^r^ Mob^+^	Simon et al. 1983
pUC4K	A plasmid conferring Amp and Km resistance	Amersham

Amp, ampicillin; Km, kanamycin; Nal, nalidixic acid; Psb, pseudobactin374; Psm, pseudomonine; Rif, rifampicin; SA, salicylic acid; Tc, tetracycline.

### Pseudomonine biosynthesis- and transport-coding region of WCS374

*Eco*RI fragments containing 12,260, and 3268 bp of the 28-kb fragment from genomic DNA of WCS374, responsible for SA and Psm biosynthesis (Mercado-Blanco et al. 2001) and transport, were cloned in plasmids pE1 and pE4, respectively, and sequenced in both directions using a primer-walking strategy (Strauss et al. 1986). Sequences were edited using DNASTAR (Lasergene) software. For homology analysis, the BLASTX and BLASTN programs at the NCBI network service were used (Altschul et al. 1997).

### Salicylic acid/pseudomonine biosynthetic mutants of WCS374

Homologous recombination was employed to inactivate genes *pmsA* or *pmsB*. Plasmids pJMsal-10, harboring a 0.7-kb *Eco*RI–*Pst*I fragment containing the *pmsB* gene, and pJMsal-20, harboring a 1.7-kb *Eco*RI – *Hin*dIII fragment containing the *pmsAB* genes (Mercado-Blanco et al. 2001), were digested with *Mlu*I and *Sal*I, respectively. A 1.2-kb Km resistance cassette from pUC4K was introduced at these sites to disrupt *pmsA* and *pmsB* (pJMsal-10::Km, and pJMsal-20::Km). Subsequently, the disrupted frames were subcloned in the broad host-range delivery suicide plasmid pSUP202, which is unable to replicate in *Pseudomonas*. The resulting recombinant plasmids, pJMpmsA::Km and pJMpmsB::Km, were used as suicide delivery vehicles to mutagenize WCS374r. Matings were performed using *E. coli* S17-1, harboring the recombinant plasmids as the donor strain. Sensitivity to Tc was used as the selection marker for colonies with frame exchange as a result of double crossing-over.

The mutants in the *pmsA* and *pmsB* genes were checked for their exchanged fragment by PCR using the primer pairs DHC01/SAL02 and SAL01/SAL02, respectively (Mercado-Blanco et al. 2001).

Cross-feeding experiments were performed to restore the production of Psm by growing *pmsA* and *pmsB* mutant strains in SSM minimal medium (Meyer and Abdallah 1978), amended with either SA (25 μg/mL) or histamine (100 μg/mL), the products of the genes involved, or with the biosynthetic precursors of SA, chorismic acid, or histamine, histidine, respectively.

To determine siderophore activity of Psm, different concentrations of the iron chelator ethylenediaminedi-O-hydroxyphenyl-acetic acid (EDDHA) (Koster et al. 1993) were added to KB agar medium. The plates were incubated at 28°C and growth of the bacteria was scored after 24 h.

### Mutants defective in the production of pseudobactin374

Strains 4A-1 (pmsA^−^) and 4B-1 (pmsB^−^) were subjected to Tn*5* mutagenesis to isolate mutants additionally defective in Psb374 production. The mobilization system of *E. coli* strain S17-1 (Simon et al. 1983) and the suicide plasmid pJQ18 (Hynes et al. 1989), which carries a modified Tn*5* (Mob Tc^r^), were used. Colonies that were nonfluorescent under UV irradiation on KB medium, indicative that Psb374 production was abolished, were selected.

### Purification of WCS374 Pseudobactin and determination of its structure

Bacteria were grown in SSM and Psb374 was extracted and purified according to Leeman et al. (1996). To avoid contamination with SA or Psm, mutant 4B-1, deficient in SA and Psm production, was used.

Alternatively, Psb374 of WCS374r and its mutants 4A-1 and 4B-1, and Pvd of strain ATCC 13525 [Pvd(13525)] were prepared according to Meyer et al. (1998). In this method, bacteria were grown in casamino acid medium (Höfte et al. 1993) and cell-free culture supernatants were passed through an XAD-4 Amberlite column (Rohm and Haas, Philadelphia, PA) to retain the fluorescent siderophores. After washing with distilled water, siderophores were eluted using 50% methanol, and lyophilized. The XAD-purified fractions were analyzed by isoelectrofocusing (IEF) according to Koedam et al. (1994).

To determine the structure of Psb374, mass spectrometric (MS) analysis was performed using a quadrupole time of flight (QToF) microinstrument (Waters, Micromass, Manchester, UK) equipped with a nano electrospray source. Samples of 10 μL were introduced into the mass spectrometer with gold-coated borosilicate capillaries at 1.2 kV. Sample cone was set at 50 V and argon was used as collision gas at ca. 20–25 V collision energy (Catalina et al. 2004). Main fragments found on mass spectra were further analyzed with tandem mass spectrometry (MSMS).

### Molecular characterization of transposon insertions

The presence of single Tn*5* insertions in SA-deficient mutants was checked by DNA–DNA hybridization. Total DNA from WCS374r and its mutants was isolated, digested with *Eco*RI, blotted onto a membrane (Hybond-N+, Amersham, Amsterdam, the Netherlands), and hybridized against a 1.2-Kb *Pst*I Km-resistance fragment excised from plasmid pUC4K, which was labeled with [α-^32^P] dCTP according to manufacturer's instruction, using high-stringency conditions (Sambrook et al. 1989).

To identify the genes that were affected in the Psb374 biosynthesis – mutants 374-02, 374-08, and double mutants AT-12 and BT-1 – the DNA sequences flanking the transposons were determined by arbitrary PCR (Caetano-Anollés 1993). A primer unique to the right end of Tn*5* elements (Tn*5*Ext, 5′-GAACGTTACCATGTTAGGAGGTC-3′) and an arbitrary primer1 (ARB1, 5′-GGCCACGCGTCGACTAGTACNNNNNNNNNNGATAT-3′) were used in the first PCR round. One microliter product of this reaction was subjected to a nested PCR reaction with primers ARB2 (5′-GGCCACGCGTCGACTAGTAC-3′) and Tn*5*Int (5′-CGGGAAAGGTTCCGTTCAGGACGC-3′). The ARB2 sequence was identical to the 5′ end of the ARB1 primer and the sequence of Tn*5*Int was identical to the right end of Tn*5,* near the insertion of the transposon in the chromosome. PCR conditions for the first round were 5 min at 95°C, 6 × [30 sec at 95°C, 30 sec at 30°C, and 1.5 min at 72°C], and 30 × [30 sec at 95°C, 30 sec at 45°C, and 2 min at 72°C]. Nested reaction conditions were 30 × [30 sec at 95°C, 30 sec at 45°C, and 2 min at 72°C]. An Eppendorf® thermocycler (Eppendorf, Nijmegen, the Netherlands) was used for the PCR reactions. The PCR products from the second reaction were subjected to agarose gel electrophoresis and purified using a DNA gel extraction kit (Millipore; Millipore corporation, Bedford, MA) as described by the manufacturer. The amplicons were sequenced using the second set of primers (nested) and compared with the GenBank DNA sequence database using the BLASTX and BLASTN Programs (Altschul et al. 1997).

### Detection and quantification of iron-regulated metabolites

To quantify SA and Psm production by WCS374r and its mutants, cells were grown in liquid SSM for 48 h. Cultures were centrifuged for 10 min at 10,000 *g*. The supernatants were filtered (0.2 μm), and aliquots of 50 μL were analyzed by HPLC. To obtain a pure reference sample of Psm, the bacterial culture supernatant was lyophilized to reduce the volume and the residue was redissolved in ultrapure water. Psm was solid-phase extracted using an Alltech C-18 reverse-phase column (Sephadex™ G-25M; Amersham Biosciences, Uppsala, Sweden), and subjected to HPLC analysis. Psm was further purified by recovering fractions from the column. The Diode array HPLC system (Shimadzu LC10AD vp; Shimadzu corporations, Kyoto, Japan) consisted of a DGU-14A degasser and a reverse-phase column of 15 cm × 4.6 mm, 5-μm pore size (SUPELCOSIL™ LC-ABZ; Sigma-Aldrich, Zwijndrecht, Netherlands), preceded by a guard column of 2 cm × 2.1, 3, and 4 mm ID guards (Supelguard™; Sigma-Aldrich, Zwijndrecht, the Netherlands). A photo diode array detector (Shimadzu SPD – M10A vp; Shimadzu, Kyoto, Japan) was used to detect and record the absorbance from 199 to 800 nm over the 35-min run. For SA and Psm analysis, a gradient of acetonitrile (HPLC grade) and 25 mmol/L KH_2_PO_4,_ pH 2.6, was used from 15% to 55% acetonitrile (0–35 min). The column was regenerated by applying 55–95% acetonitrile (35–38 min), 95–55% acetonitrile (38–42 min), 55–15% acetonitrile (42–45 min), and 15% acetonitrile (45–55 min); the flow rate was 1 ml/min. Absorbances of SA and Psm were specifically monitored at 210, 236, and 295 nm, and areas under the recorded peaks were analyzed using software provided by the manufacturer (Class-VP). To quantify the SA and Psm present in the culture supernatants, different amounts of pure SA and Psm were dissolved in SSM. Fifty microliters of culture supernatant were injected and compared with a standard range of known concentrations.

To further characterize the mutants with no or reduced production of free SA*,* samples of filtered culture supernatant, or fractions collected from the HPLC, were lyophilized and subjected to MS measurements as described above.

The amounts of Psb374 produced in SSM liquid cultures were determined spectrophotometrically at 400 nm and expressed on the basis of the number of cells at the time of harvesting.

### Plant growth and colonization of Arabidopsis roots by WCS374r and its mutants

Seeds of *Arabidopsis thaliana* accession Col-0 were sown in sterile quartz sand and seedlings were grown for 2 weeks. Plastic pots (60 ml) were filled with a mixture of sand and potting soil (5:12) that was autoclaved twice for 1 h with a 1-day interval, and three seedlings were transplanted into each pot. Bacterial strains were incubated at 28°C for 24 h on KB agar plates. Cells were scraped off the plates and suspended in sterile 10 mmol/L MgS0_4_. Before transferring the seedlings, the sand–potting soil mixture was supplemented with a suspension of bacteria to a final density of 10^3^ colony forming units (cfu) per gram soil. Plants were watered on alternate days and once a week supplied with a modified half-strength Hoagland nutrient solution (Hoagland and Arnon 1938) without iron. Plants were cultivated in a growth chamber with an 8-h day (200 μE/m^2^/s^1^ at 24°C) and a 16-h night (20°C) cycle at 70% relative humidity.

One, two, and three weeks after transplanting, roots of each treatment were sampled, weighed, rinsed briefly in water, and shaken vigorously for 1 min in glass tubes containing 5 ml 10 mmol/L MgSO_4_ and 0.5 g of glass beads (0.17-mm diameter). Aliquots of appropriate dilutions were plated on KB agar supplemented with cycloheximide (100 μg/mL), ampicillin (50 μg/mL), chloramphenicol (13 μg/mL) [KB^+^] (Geels and Schippers 1983), and rifampicin (150 μg/mL) (Glandorf et al. 1992), or kanamycin and streptomycin (100 μg/mL) (Bakker et al. 1988). After overnight incubation at 28°C, the number of colonies was counted and cfu per gram of root fresh weight were calculated. Treatments consisted of nine pots for each time point. Experiments were performed twice with similar results.

Populations of bacterial cells on plant roots approximate a log-normal distribution (Loper et al. 1984). Therefore, values were submitted to logarithmic transformation prior to one-way analysis of variance (ANOVA), followed by Fisher's test for least-significant differences at *α* = 0.05.

### Induced systemic resistance bioassays

*Arabidopsis thaliana* accession Col-0 was grown as described by Pieterse et al. (1996). Seeds were sown in quartz sand and 2-week-old seedlings were transferred to 60-ml pots containing a sand–potting soil mixture that was autoclaved twice for 20 min with a 24-h interval. The soil was inoculated by mixing the bacterial suspension to final densities of 10^3^, or 5 × 10^7^ cfu/g soil prior to transplanting the seedlings. Plants were cultivated in a growth chamber with an 8-h day (200 μE m^−2^ s^−1^ at 24°C) and 16-h night (20°C) cycle at 70% relative humidity. Plants received half-strength Hoagland nutrient solution (Hoagland and Arnon 1938) containing 10 μmol/L Sequestreen (CIBA-Geigy, Basel, Switzerland) at the time of transplanting and were watered on alternate days. The bacterial pathogen *P. syringae* pv. *tomato* DC3000 (Pst) was cultured overnight at 28°C in liquid KB medium. Bacterial cells were collected by centrifugation and resuspended in 10 mmol/L MgSO_4_ containing 0.0125% (v/v) Silwet L-77, to a final density of 5 × 10^6^ cfu/mL. Five-week-old Arabidopsis plants were challenge inoculated by dipping the leaves into the Pst suspension. Three to four days later, the percentage of leaves with symptoms was determined per plant. Leaves showing necrotic or water-soaked lesions surrounded by chlorosis were scored as diseased. For each treatment 20–25 plants were used. Results were submitted to ANOVA, followed by Fisher's test for least-significant differences at *α* = 0.05.

## Results

### Salicylic acid and pseudomonine biosynthesis genes in WCS374

We corroborated the organization of the SA and pseudomonine biosynthesis genes in WCS374 that was previously reported (Matthijs et al. 2009). The SA biosynthesis genes in WCS374 are organized in a cluster designated *pmsCEAB*, that was cloned previously as pE3 (Mercado-Blanco et al. 2001). *pmsCEAB* is part of a 28-kb fragment, of which pE4 (3268 bp) and pE1 (12260 bp) were sequenced and found to contain 10 additional open reading frames (ORFs) that are presumably involved in Psm biosynthesis and transport (Genbank accession number AN: EF484930). Of these, *pmsD*, *pmsF,* and *pmsG* complete the Psm biosynthesis machinery (Sattely and Walsh 2008; Matthijs et al. 2009; Wuest et al. 2009).

### Salicylic acid and pseudomonine mutants of WCS374r

Site-directed mutagenesis was employed to disrupt the *pmsA* or the *pmsB* gene in WCS374r. Mutants were recovered and identified on the basis of their resistance to kanamycin.

Selected mutants yielded a single PCR amplicon with the primer pairs DHC01/SAL02 and SAL01/SAL02 (Mercado-Blanco et al. 2001). As expected, the PCR products from mutants in *pmsA* and *pmsB* were about 1 kb larger than the amplicons from the native genes because of the presence of Km^R^ cassette within the genes (data not shown). These results, together with the criterion used for selecting transconjugants (Km resistance and Tc sensitivity), confirmed that gene replacement had occurred as a result of double crossing-over events between recombinant plasmids pJMpmsA::Km and pJMpmsB::Km, and *pmsA* and *pmsB* native genes, respectively. Southern blot analysis revealed the presence of an insert in the *pmsA* and *pmsB* mutants, which was not present in wild-type WCS374r, when a 1.2-Kb *Eco*RI fragment of pUC4K was used as a probe for Km^r^ cassette (data not shown). 4A-1 and 4B-1 were selected as representative mutants in genes *pmsA* and *pmsB*, respectively. Table 2 summarizes the biosynthetic capacities of the mutants to produce SA, Psm, and Psb374. Mutants 4A-1 and 4B-1 did not produce Psm and SA/Psm, respectively, in SSM minimal medium, but produced more Psb374 compared with the wild type.

**Table 2:** Pseudobactin, SA, and pseudomonine production by *Pseudomonas fluorescens* WCS374r and its mutants

Strain	Pseudobactin (fg/cell)	SA (fg/cell)	Pseudomonine(fg/cell)
WCS374r	153.2 ± 5.1 C*	100.0 ± 9.2 A	290 ± 11.2 C
374-02	ND	50.0 ± 3.4 B	380 ± 8.2 B
374-08	ND	50.1 ± 8.1 B	420 ± 10.8 A
4B-1	234.5 ± 4.7 B	ND	ND
4A-1	455.5 ± 21 A	5.3 ± 5.1 D	ND
BT-1	ND	ND	ND
AT-12	ND	10.0 ± 2.0 C	ND

The values presented are the averages from at least three independent experiments.

* Within each column values with different letters are significantly different (*P* = 0.05). ND, not detected.

### Pseudobactin374 mutants in the salicylic acid/pseudomonine mutant background

To generate mutants defective in both Psm and Psb374 biosynthesis, Tc-resistant colonies were obtained after conjugation of mutants 4A-1 and 4B-1 with donor strain *E*. *coli* S17-1 carrying the Tn*5* transposon (pJQ18). Colonies that were nonfluorescent on KB medium, indicating that Psb374 production was abolished, were selected. Selected mutants were designated as WCS374-AT and -BT strains when they carried a mutation in Psb374 production in the 4A-1 or 4B-1 background, respectively. Of the resulting double mutants, AT-12 and BT-1 were selected for further analysis. With regard to SA and Psm synthetic capacity, AT-12 and BT-1 were similar to their parent strains.

The DNA sequences flanking the Tn*5* insertions in the Psb374 mutants were characterized. The deduced amino acid sequence of the translation product of the sequence flanking the insertion in strain AT-12 (300 bp) showed 60% identity to pyoverdine synthetase A of a *P*. *fluorescens* strain (Mossialos et al. 2002) (AN: AAF40219, similarity 75%), 55% identity to pyoverdine chromophore precursor synthetase of *P. syringae* pv. *phaseolicola* 1448A and *P. syringae* pv. *syringae* DC3000 (AN:YP_274139.1, similarity 74%, and NP_791957.1, similarity 74%, respectively), and 56% identity to NRPS modules of *P. fluorescens* Pf-01 (AN: ZP_00262446.1, similarity 74%). A 100% identity at the nucleotide level (395 bp) with *pvdS* of *P. fluorescens* (Miyazaki et al. 1995) was found for the flanking region of Tn*5* in the strain BT-1. The deduced translated product of this fragment showed 32% identity and 39% similarity to putative metal cation transporter ATPases of *Mycobacterium tuberculosis* strains H37Rv and F11 (AN: NP_215423.1, and AN: EAX12793.1, respectively).

Two Tn*5* mutants of WCS374r lacking Psb374 only were similarly characterized. A 250-bp amplicon obtained from mutant WCS374-02, showed 94% identity to a nucleotide sequence of pyoverdine synthetase (*pvdS*) in *P*. *fluorescens* (AN: AF237701). Other similarities were found with a putative pyoverdine chromophore precursor synthetase from *P. syringae* pv. *phaseolicola* 1448A (91% identity, AN: CP000058), and putative pyoverdine synthetase from *P*. *putida* KT2440 (88% identity, AN: AE016789). At the amino acid level, best homologies were found with iron-sulfur binding proteins of *Kineococcus radiotolerans* SRS30216 (AN: EAM74978.1 and ZP_00617246.1; Identity 30%, similarity 41%, in 80 amino acids aligned). A 400-bp amplicon was obtained from Psb374 mutant 374-08 and showed best homology at the protein level with NRPS modules of *P. fluorescens* Pf-01 (AN: ZP_00265633.1; Identity 44%, similarity 57%, in 56 amino acids aligned). Other similarities were found with an NRPS of *P. fluorescens* Pf-05 (AN: YP_261192.1; Identity 39%, similarity 55%, in 56 amino acids aligned) and *P. putida* KT2440 (AN: NP_746338.1; Identity 39%, similarity 51%, in 56 amino acids aligned).

### Purification of Pseudobactin 374 and determination of its structure

Based on IEF patterns, homology between Psb374 of *P*. *fluorescens* strains WCS374r and Pvd(13525) was suggested (Fuchs et al. 2001). Therefore, the purified Psb374 of WCS374r and its mutants 4A-1 and 4B-1 were compared to that of ATCC 13525 using IEF. In agreement with Fuchs et al. (2001), purified Psb374 and Pvd13525 showed identical IEF patterns (data not shown). Moreover, mutants 4A-1 and 4B-1 presented patterns identical to wild-type WCS374r with one band at pI 8.7 and two bands around 7–7.2, indicating that they produce the same Psb374 molecules (data not shown).

**Figure 1:**
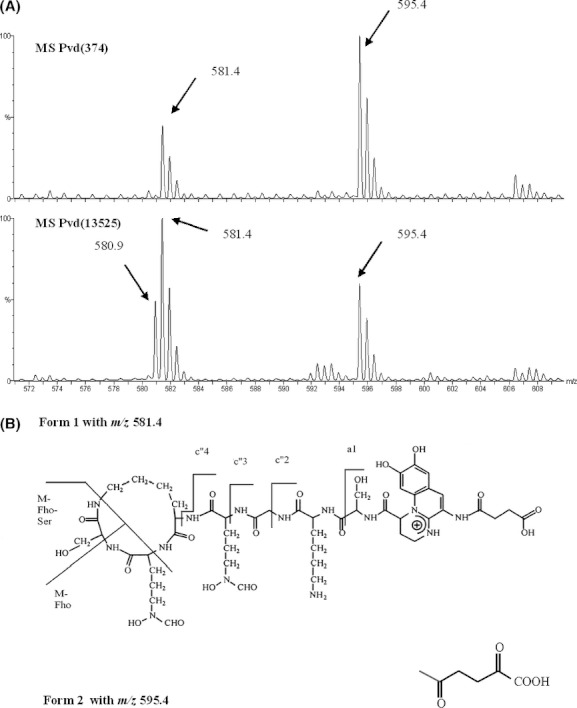
Molecular characterization of Psb374 in comparison to Pvd13525. (A) Mass spectra of the doubly charged fragment ions from Psb374 (above), and Pvd of ATCC 13525 (below), and (B) structure of the Psb374 isoforms with fragmentations assigned after mass spectrometric analysis. Form 1 represents the succinic acid-Psb, and form 2 represents the ketoglutaric acid-Psb. Picture of Psb is adapted from Fuchs et al. (2001). Fragmentations indicated in form 1 are assigned as detailed in Table 3.

The molecular structure of Pvd(13525) is well known (Fuchs et al. 2001; Schäfer et al. 2006). Mass spectrometric analysis of purified Psb374 and Pvd13525 revealed that they contain fragments with the same molecular weights; however, their ratios were different. Two main peaks with *m/z* of 595.4 and 581.4 were present in the MS spectra of these compounds (Fig. 1A). In the MS spectrum of Psb374, the doubly charged ion (M + 2H)^2+^ of *m/z* 595.4 was dominating, whereas in the MS spectrum of Pvd13525, the one with a (M + 2H)^2+^ of *m/z* 581.4 was the major compound (Fig. 1A). In addition, a (M + 2H)^2+^ of *m/z* 580.9 in the Pvd13525 was observed, which was absent in the spectrum of Psb374 (Fig. 1A). MSMS analysis showed that the fragmentations of the peak with *m/z* 581.4 in both samples are identical (data not shown). A fragment ion at *m/z* 417 indicates the presence of a succinic acid residue (Table 3). The MSMS spectra of *m/z* 595.4 of both samples were also similar (data not shown). The fragmentations of these compounds were preceded by a loss of CO_2_ and H_2_O from the acyl side chain of the chromophore. Comparison of the mass of this fragment ion with the mass of putative acyl side chains in chromophores of other Pvd molecules (Fuchs et al. 2001) suggested that this fragment is ketoglutaric acid (Kgl). Furthermore, consistent with Schäfer et al. (2006), the MSMS spectrum of *m/z* 580.9, which was only present in Pvd13525, revealed a fragment ion at *m/z* 416, indicating the presence of a succinic acid amide side chain in this strain (data not shown).

**Table 3:** Product ions of fragments with *m/z* 581.4 and 595.4 from Psb374. Values corresponding to fragmentations of *m/z* 595.4 are after subtraction of one mass equal to a (CO_2_ + H_2_O). Fho: *N*-formyl-*N*-hydroxy ornithine; M: Molecule; Ser: serine; a and c” represents ions with the charge retained on the N-terminal fragment (Roepstorff and Fohlmann 1984)

Assignment	*m/z* 581.4 (+succinic acid)	*m/z* 595.4 (+ketoglutaric acid)
a1	417.3	383.3
c”2	590.5	556.5
c”3	647.5	613.5
c”4	805.7	771.7
M-Fho	1003.9	969.9
M-Fho-Ser	916.8	882.8

The monoisotopic molecular weight of Psb374 was determined to be 1160.53 and 1188.53 for Psb374-succinic acid and Psb374-Kgl, respectively. The molecular weight of Psb374-Kgl was used for the quantification of Psb374 in the experiments. For ATCC 13525, the monoisotopic molecular weight of the succinic acid-amide-Pvd was determined to be 1159.53, which is identical to that described previously (Schäfer et al. 2006).

MSMS analysis of other fragment ions present in the mass spectra, revealed that the peptide chain and the chromophore of Psb374 and Pvd13525 are identical (data not shown). Based on the results of MS and MSMS analyses, the structure of Psb374 was characterized (Fig. 1B). The Psb374 consists of two siderophores, which are different only in the acyl side chain.

A calibration curve for pure Psb374 was produced and used for quantification of Psb374 production by WCS374r and its mutants in further experiments.

### Determination and quantification of salicylic acid and pseudomonine

To simultaneously quantify SA and Psm production by WCS374r and its mutants, the HPLC method of Meuwly and Métraux (1993) was adapted. Psm was first extracted by solid-phase extraction from WCS374r culture supernatants according to Mercado-Blanco et al. (2001), and fractions were collected from several HPLC runs. MS analysis confirmed that the collected peak corresponded to Psm. In addition, MSMS of the parent ion with *m/z* 331 showed the same fragmentation profile as described before (Mercado-Blanco et al. 2001). Culture supernatants from mutants 4A-1 and 4B-1 were also subjected to mass spectrometry. Absence of a molecule with mass 331 was established, whereas culture supernatants of Psb374 mutant 374-08 did show this molecule. Major peaks in the MS spectra of culture supernatants of 4A-1 and 4B-1 were examined by MSMS analysis as well, but did not correspond to any residual fragments of the Psm molecule (data not shown).

For quantification by HPLC, culture supernatants were analyzed directly without prior purification. Upon analysis, peaks corresponding to Psm and SA appeared at 5.7 and 25.6 min, respectively. Table 2 summarizes the levels of SA and Psm in cell-free supernatant of WCS374r, and its various mutants.

The Psb374 mutants 374-02 and 374-08 produced less SA, but more Psm than WCS374r (Table 2). As expected, mutant 4B-1 and its Psb374 derivative BT-1 failed to synthesize SA and Psm, whereas mutant 4A-1 and its Psb374 mutant AT-12 synthesized a small amount of SA, but no Psm. SA quantities produced by 4A-1 and AT-12 were about 5–10% of the amounts produced by wild-type WCS374r (Table 2).

### PmsA and PmsB activities are essential for biosynthesis of pseudomonine

**Figure 2:**
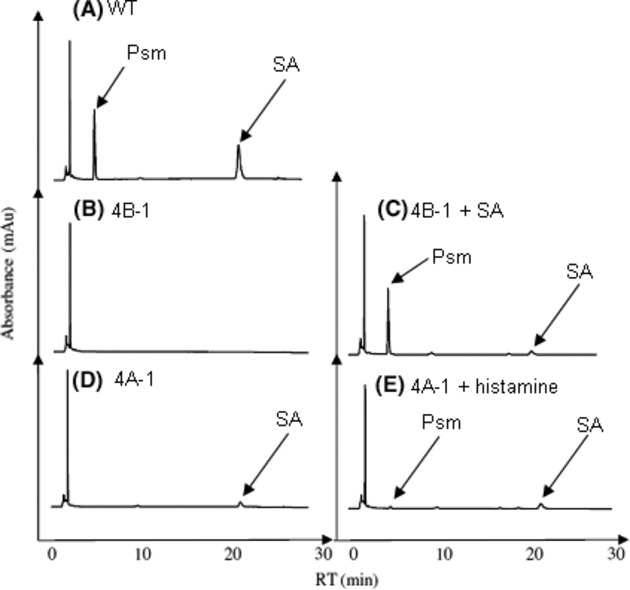
HPLC chromatograms of culture supernatants of (A) WCS374r, (B, C) mutant 4B-1, and (D, E) mutant 4A-1 grown in SSM medium in the absence (B, D), or presence of (C) SA (25 μg/ml), or (E) histamine (100 g/ml).

Cross-feeding experiments were performed to restore Psm production in the *Psm* mutants. HPLC analysis showed that mutants 4A-1 and 4B-1 produced Psm when histamine (100 μg/mL) and SA (25 μg/mL), respectively, were added to the SSM minimal medium (Fig. 2). The production of Psm in 4A-1 was restored by adding histamine, and in 4B-1 Psm production was restored by adding SA. Addition of precursor substrates L-histidine (5 mg/ml), or chorismic acid (500 ng/ml), did not result in restoration of Psm production (data not shown). These results demonstrate that the function of the genes *pmsA* and *pmsB* is necessary for the biosynthesis of Psm by WCS374.

### Pseudomonine is a physiologically active siderophore of WCS374

Biological evidence for the role of Psm as a molecule with siderophore activity was provided by adding different concentrations of EDDHA, a strong iron chelator, to KB agar medium prior to inoculation of the bacterial strains. Results are presented in Table 4. The parental strain WCS374r did grow on concentrations up to 5 mmol/L EDDHA. Loss of Psm production in mutants 4A1 and 4B1 did not affect its ability to grow on high concentrations of EDDHA. In contrast, the growth of Psb374 mutants 374-02 and 374-08 was inhibited at 500 μmol/L EDDHA. In the Psb374 background, production of Psm did affect growth on EDDHA as the Psb374/Psm double mutants AT-12 and BT-1 did not grow anymore at a concentration of 100 μmol/L, indicating that Psm is an iron-scavenging molecule for WCS374r. These data suggest that Psb374 is the main siderophore of WCS374r and that Psm contributes when Psb374 production is lacking.

**Table 4:** Growth of WCS374 and its iron-regulated deficient derivatives on KB agar amended with different concentrations of EDDHA (μmol/L). +: growth −: no growth

	EDDHA concentration (μmol/L)					
	
	0	50	100	500	1000	5000
WCS374	+	+	+	+	+	+
Psb^−^	+	+	+	−	−	−
Psm^−^	+	+	+	+	+	+
Psm^−^/Psb^−^	+	+	−	−	−	−

### Colonization of Arabidopsis roots by WCS374r and its mutants

**Figure 3:**
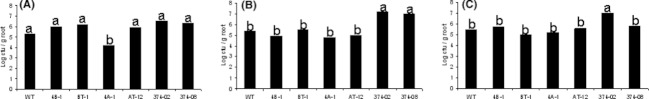
Root colonization of Arabidopsis by WCS374r (WT) and pseudomonine (4B-1 and 4A-1), pseudobactin374 (374-02 and 374-08), and double mutants (BT-1 and AT-12). Root samples were collected after 1 (A), 2 (B), and 3 (C) weeks after transplanting seedlings into soil inoculated with the different WCS374 derivatives. Different letters indicate statistically significant differences between treatments (Fisher's least-significant difference test, *α* = 0.05).

The significance of iron-regulated metabolites in colonization of Arabidopsis roots by WCS374r was assessed. WCS374r and its various mutants were introduced into the soil at a density of 10^3^ cells per gram, and colonized the roots to levels at least equal to the wild type (Fig. 3).

### *P. fluorescens* WCS374-mediated ISR against *P. syringae* pv. *tomato* in Arabidopsis depends on WCS374r inoculum density

**Figure 4:**
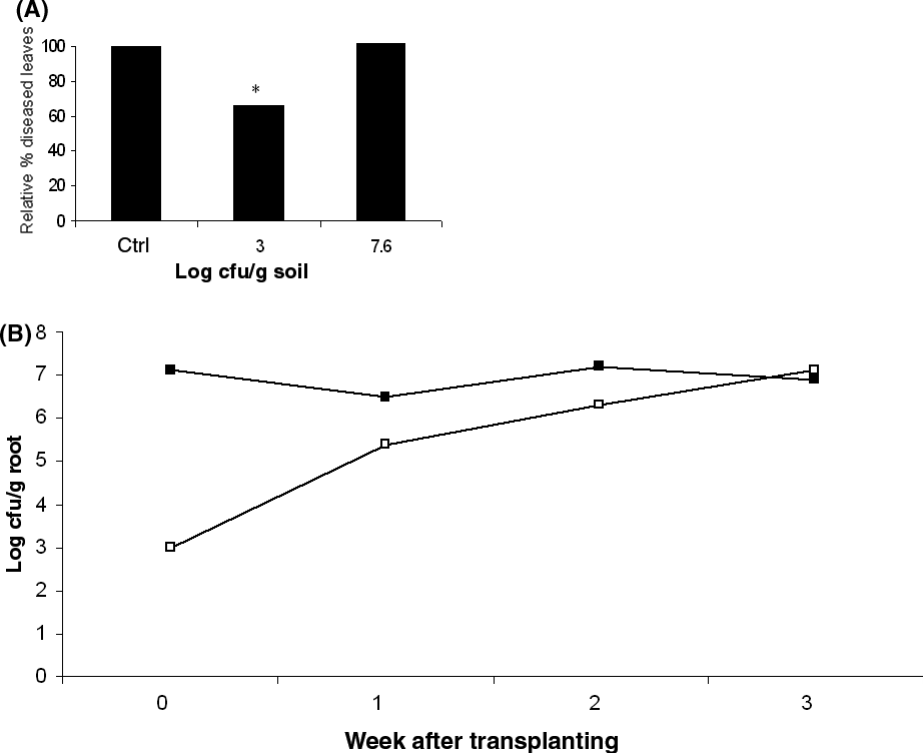
ISR-eliciting activity of WCS374 in Arabidopsis. (A) Relative percentage of diseased leaves by *Pseudomonas syringae* pv. *tomato* DC3000 was reduced when WCS374r was applied at low inoculum density, but not at high inoculum density. Symptoms were scored 3 days after challenge. An asterisk indicates a statistically significant difference (Fisher's least-significant difference test; *α* = 0.05, *n* = 20–25). (B) Population densities of WCS374r in Arabidopsis rhizosphere after introduction of the bacterium into the soil at a density of 10^3^ (□) or 5 × 10^7^ (▪) cfu/g.

As reported earlier by Van Wees et al. (1997) application of WCS374r at a density of 5 × 10^7^ cfu/g soil did not raise the resistance of colonized plants; however, when applied at a dose of 10^3^ cfu/g soil, WCS374r significantly reduced the symptoms of bacterial infection by Pst (Fig. 4A), demonstrating that this strain triggers ISR against this pathogen in Arabidopsis. After 3 weeks, population densities of WCS374r on Arabidopsis roots were around 10^7^ per gram, independent of the initial density applied to the soil (Fig 4B). Thus, whereas cells applied at the high density had not increased, those applied at the low densities must have multiplied actively.

### Iron-regulated metabolites of WCS374 do not play a role in ISR against Pst in Arabidopsis

**Figure 5:**
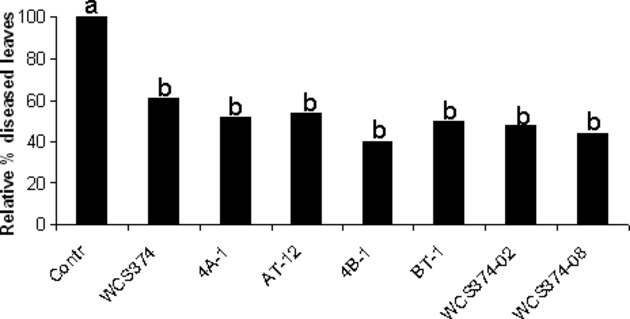
Involvement of iron-regulated metabolites in WCS374-mediated ISR in Arabidopsis against *Pseudomonas syringae* pv. *tomato* DC3000. Relative disease incidence in plants treated with WCS374r or its mutants in the production of iron-regulated metabolites is shown. Different letters indicate statistically significant differences between treatments (Fisher's least-significant difference test; *α* = 0.05).

Strain 4B-1 is deficient in the production of both SA and pseudomonine, and was shown to be as effective as the wild type against Pst (Fig. 5). To unravel the role of SA biosynthesis, strain 4A-1, a constitutive producer of SA, was used. Furthermore, other mutants additionally defective in Psb374 were also included (Table 1). As shown in Figure 5, all mutants defective in the production of iron-regulated metabolites significantly reduced the relative percentage of diseased leaves compared to non-induced plants by 40–60%. This finding indicates that neither the biosynthesis of SA, nor Psm, nor Psb374 is required for triggering ISR against Pst. All mutants colonized the roots at least as well as the wild-type strain. These results suggest that WCS374 elicits ISR through other traits.

## Discussion

Pyoverdine is considered to be the primary siderophore involved in the uptake of iron by fluorescent pseudomonads. The structure of pyoverdines is variable among *Pseudomonas* species and even between strains of some species (Meyer et al. 2002; Visca et al. 2007). Close to 70 structurally different pyoverdine molecules have been identified so far (Meyer et al. 2008). To elucidate the molecular structure of the fluorescent siderophore produced by the plant growth-promoting rhizobacterium (PGPR) strain *P. fluorescens* 374r, we used Pvd13525 as a reference, as based on their IEF patterns, Psb374 is similar to Pvd13525 (Fuchs et al. 2001). In agreement with Fuchs et al. (2001) and Schäfer et al. (2006), Pvd13525 showed three major peaks when subjected to MS analysis, corresponding to pseudobactins with either a ketoglutaric acid, a succinic acid, or a succinic amide acyl side chain of the chromophore. Psb374 showed two major peaks corresponding to pseudobactins with a ketoglutaric acid or succinic acid acyl chain. Despite the similarity in structure of Psb374 and Pvd13525, that is pseudobactin-ketoglutaric acid and pseudobactin-succinic acid, their relative abundances were different, and the dominating molecule of WCS374 was the ketoglutaric acid variant.

Many bacteria produce more than a single type of siderophore (Jones and Wildermuth 2011). For WCS374r, production of an additional siderophore, besides Psb374 and SA, was suggested (Leeman et al. 1996). Mercado-Blanco et al. (2001) showed that the additional siderophore is Psm, an isoxazolidone-containing histamine, cyclothreonine and SA. These authors demonstrated that SA and Psm biosynthesis genes are closely linked in the recombinant clone pMB374-07. Only 5 kb of pMB374-07, containing four ORFs and designated as *pmsCEAB*, were required for SA production in *E. coli*.

We mutated genes *pmsB* (encoding isochorismate pyruvate-lyase), and *pmsA* (encoding histidine decarboxylase) in WCS374 to determine the significance of these genes in Psm biosynthesis. Disruption of *pmsB* resulted in a complete lack of SA and Psm production (Table 2). Feeding of mutant 4B-1 with SA restored Psm biosynthesis, indicating that *pmsB* is necessary for the cells to produce SA.

Histidine decarboxylase activity of the product of *PmsA* was similarly investigated by feeding experiments. Mutant 4A-1 did not produce Psm, but still produced 5% of the amount of SA produced by the wild type (Table 2). Feeding 4A-1 with histamine partially restored Psm biosynthesis, but adding the putative precursor substrate of this enzyme, histidine, to the minimal growth medium did not result in Psm accumulation. Partial restoration of Psm production upon addition of histamine could be explained by the low levels of SA produced by 4A-1. Based on these results, *pmsA* appears to be necessary for the synthesis of the histamine moiety of Psm.

Siderophore activity of Psm in *P. fluorescens* WCS374r was studied by growing the various mutants on media amended with different concentrations of the chelator EDDHA. Compared with the Psb374 mutants, Psb374/Psm double mutants were even more impaired in their growth on EDDHA (Table 4), indicating that Psm has siderophore activity in WCS374r. Moreover, Psm mutants produced higher levels of Psb374 compared with the wild type (Table 2), possibly to compensate for the lack of the additional siderophore in iron-poor conditions.

Previously, WCS374 was reported not to be able to elicit ISR in Arabidopsis (Van Wees et al. 1997). Indeed, in agreement with Van Wees et al. (1997), high inoculum densities (5 × 10^7^ cfu/g of soil) of WCS374 failed to elicit ISR in Arabidopsis against Pst (Fig. 4A). To study the root colonizing ability of WCS374r and its mutants, they were applied at low inoculum densities (10^3^ cfu/g of soil). In these experiments, we discovered that low inoculum densities of WCS374 trigger ISR against *P*. *syringae* pv. *tomato* DC3000 in Arabidopsis (Fig. 4A). When applied at such low densities, populations increased, whereas in high-density applications, the populations remained at the same level throughout the experiment. These results suggest that active growth of strain WCS374 is a prerequisite for triggering ISR in Arabidopsis.

To study the involvement of the various iron-regulated metabolites produced by WCS374 in ISR, the wild-type strain and mutants were compared in ISR bioassays. All the mutants in the biosynthesis of iron-regulated metabolites were shown to be as effective as WCS374 in mediating resistance against *P. syringae* pv. *tomato* DC3000 (Fig. 5), suggesting that these metabolites are not required in this interaction. However, redundancy of ISR-eliciting traits in *Pseudomonas* spp. has been reported (Bakker et al. 2003; Meziane et al. 2005), and thus the individual traits investigated in this study may play a role. In rice, pseudobactin of WCS374 is the crucial determinant in ISR against the leaf blast pathogen *Magnaporthe oryzae* (De Vleesschauwer et al. 2008). But in radish, lipopolysaccharides (Leeman et al. 1995) and iron-regulated metabolites (Leeman et al. 1996) are involved in ISR elicited by WCS374r. Thus, the involvement of individual elicitors in induced resistance by this *P. fluorescens* strain seems to be plant species specific.
